# Diagnosis and Rescue of a Kinked Pulmonary Artery Catheter

**DOI:** 10.1155/2015/567925

**Published:** 2015-05-13

**Authors:** Nicolas J. Mouawad, Erica J. Stein, Kenneth R. Moran, Michael R. Go, Thomas J. Papadimos

**Affiliations:** ^1^Department of Surgery, The Ohio State University Wexner Medical Center, 410 West 10th Avenue, Columbus, OH 43210, USA; ^2^Department of Anesthesiology, The Ohio State University Wexner Medical Center, 410 West 10th Avenue, Columbus, OH 43210, USA

## Abstract

Invasive hemodynamic monitoring with a pulmonary catheter has been relatively routine in cardiovascular and complex surgical operations as well as in the management of critical illnesses. However, due to multiple potential complications and its invasive nature, its use has decreased over the years and less invasive methods such as transesophageal echocardiography and hemodynamic sensors have gained widespread favor. Unlike these less invasive forms of hemodynamic monitoring, pulmonary artery catheters require an advanced understanding of cardiopulmonary physiology, anatomy, and the potential for complications in order to properly place, manage, and interpret the device. We describe a case wherein significant resistance was encountered during multiple unsuccessful attempts at removing a patient's catheter secondary to kinking and twisting of the catheter tip. These attempts to remove the catheter serve to demonstrate potential rescue options for such a situation. Ultimately, successful removal of the catheter was accomplished by simultaneous catheter retraction and sheath advancement while gently pulling both objects from the cannulation site. In addition to being skilled in catheter placement, it is imperative that providers comprehend the risks and complications of this invasive monitoring tool.

## 1. Introduction

The flow-directed balloon-tipped pulmonary artery catheter (PAC), also known as the Swan-Ganz catheter, has been in widespread clinical use for over 40 years. In fact, for patients in the United States, as many as 1 million PACs and 5 million central venous catheters are placed annually [1]. Invasive hemodynamic monitoring with the PAC has been relatively routine in cardiovascular and complex surgical operations as well as in the management of critical illness. Although the American College of Cardiology published a consensus statement in 1998 regarding the use of the PAC in cardiac disease, there are no validated indications for its general use.

The advent of more noninvasive methods to determine cardiovascular status has led way to a decrease in the use of the PAC, however, and more junior physicians are less experienced in the management of PACs and their potential complications. A recent study by Koo et al. evaluating over 15,000 patients over a 5-year period noted that 12.8% had a PAC placed; over the same study period, the adjusted rate of use of the PAC decreased from 16.4% to 6.5% [2]. The determinants of PAC use did not change over time, but the largest nonpatient related factor for PAC use was the attending physician's base specialty and intensive care unit status.

With such widespread use, it is imperative on the part of the provider to be well versed in the technique, possible complications, and rescue maneuvers should they be necessary. Perforation of the access vessels leading to hemothorax or perforation of the pleura with subsequent pneumothorax is a known complication. Perforation of the aorta with ensuing cardiac tamponade can occur if the cannula-site perforation is within the pericardial sac; this is usually associated with a mortality of 90% [3]. Arrhythmias constitute the most common complication in relation to PAC insertion. These are usually premature ventricular contractions or nonsustained ventricular tachycardia that usually resolve by retracting the catheter back into the right atrium from the right ventricle or advancing it in the pulmonary artery. Right bundle branch block is another complication and, in patients with existing left bundle branch block, care must be taken to avoid precipitating complete heart block. Necessary adjunctive medications and a temporary pacemaker should be kept nearby in such situations. Cardiac damage and valvular and structural heart injury as well as pulmonary artery vessel injury with resultant hemoptysis are also devastating potential complications.

Here, we describe the case of a patient with a PAC in which significant resistance was encountered during removal attempts due to kinking and twisting of the catheter tip and present rescue options for such a situation.

## 2. Case Description

A 57-year-old African American woman with a longstanding history of dilated cardiomyopathy secondary to mitral insufficiency was evaluated for a mitral valve repair and MAZE procedure at an outside hospital (OSH). Preoperative hemodynamic monitoring was instituted with a single stick left transinternal jugular PAC placed in the standard fashion as well as a left radial arterial line. The PAC was easily advanced using pressure curves and secured for periodic evaluation of pulmonary capillary wedge pressure. On induction of anesthesia at the OSH she had severe hypotension with pulseless electrical activity for 15 minutes. She was resuscitated with multiple boluses of epinephrine and subsequent infusions of norepinephrine, milrinone, and amiodarone. Median sternotomy was performed emergently; however the procedure was aborted and an intra-aortic balloon pump (IABP) was placed for supportive care and resuscitation. The sternotomy was then closed without placing the patient on cardiopulmonary bypass and she was transferred to our tertiary level care center for critical care management and a mitral valve repair.

On arrival, the norepinephrine was discontinued and she was placed on a dobutamine infusion; milrinone and amiodarone were continued. Preoperatively her transesophageal echocardiogram demonstrated severe systolic dysfunction with an ejection fraction of <20%, a normal sized right ventricle with moderately reduced systolic function, an enlarged left atrium, and severe mitral regurgitation with a thickened mitral valve. Six days after admission she successfully underwent a redo sternotomy with repair of the mitral valve using a 26 mm Edwards annuloplasty band. She arrived in the intensive care unit with infusions of dobutamine, milrinone, and amiodarone and the IABP still in place.

**Figure 1:**
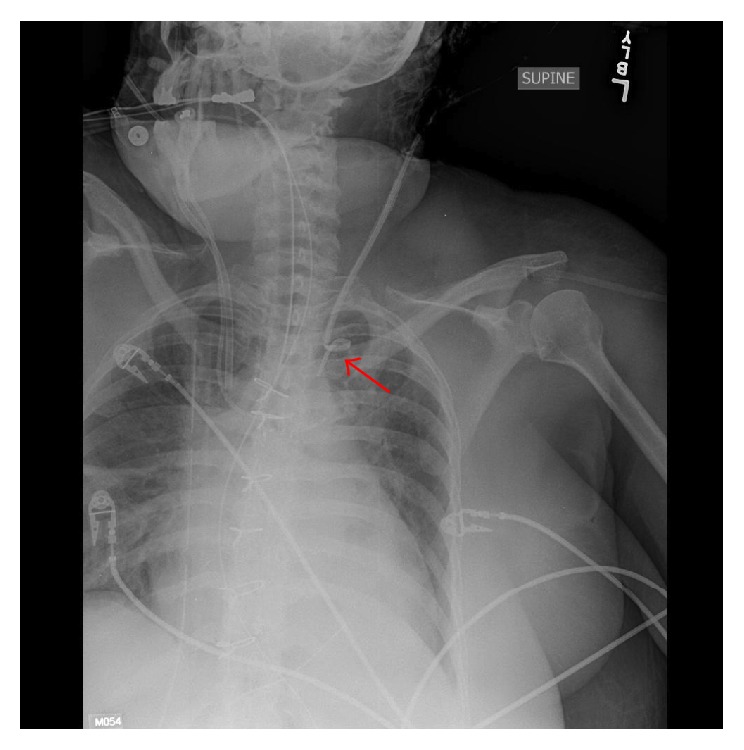
Chest radiography of kinked PAC at the confluence of the left internal jugular vein and left subclavian vein.

Two days after surgery the drips were discontinued and the IABP was removed. That same afternoon the decision was made to remove the PAC from her left internal jugular vein. After appropriate positioning, sterile technique, and assurance that the catheter balloon was deflated, the catheter sleeve was disconnected from the introducer sheath. A slow, smooth, and steady motion was employed to pull back the catheter and met with significant resistance. Two subsequent attempts at pulling and advancing the catheter were attempted to no avail, suggesting a problem. Chest radiography at the bedside was performed which demonstrated kinked PAC at the confluence of the left internal jugular vein and left subclavian vein (Figure 1). The chest roentgenogram from that same morning demonstrated that the pulmonary artery catheter was in proper position without any kinks. Vascular surgery was consulted for recommendations regarding its removal.

**Figure 2:**
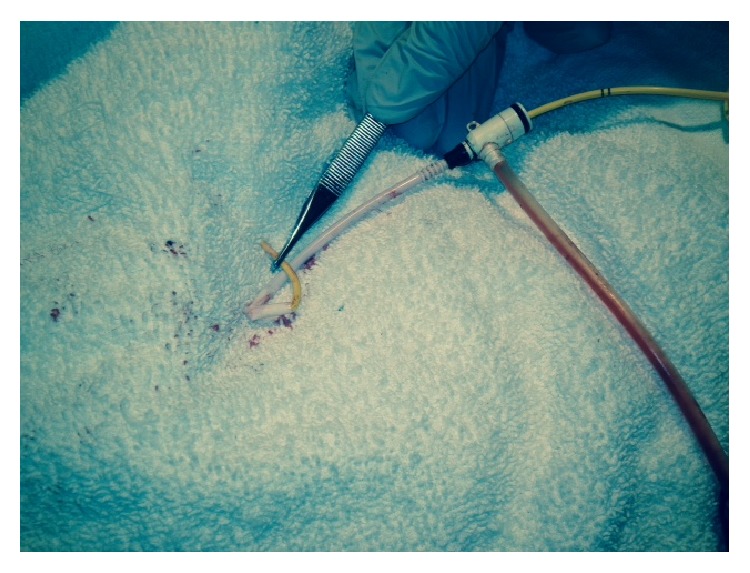
Ex vivo photograph of the PAC maintaining its kinked position.

After review of the chest X-ray, gentle initial traction was applied in an attempt to remove the catheter; significant resistance was again noted. A hybrid operating room was prepared in order to pass a semistiff or stiff guide wire to help straighten out the catheter. While awaiting transfer to the operating room the decision was made to attempt to deepen the sheath over the catheter to help straighten it out. Ultimately, with a combination of steady backward traction and forward advancement of the sheath, the catheter was retracted into the sheath allowing for a successful and safe recovery. Once the sheath was removed, the coil pattern, or kinking, of the catheter was evident and reproducible (Figure 2).

Hemostasis was achieved with a manual compression. The patient tolerated the procedure well and ultimately was discharged from the hospital to a skilled nursing facility for convalescence.

## 3. Discussion

Although the use of the PAC has decreased over time and given way to more noninvasive methods such as transesophageal echocardiography and infrared sensors that evaluate stroke volume variation and cardiac output, it still remains a well-established tool in the management of critically ill patients [2, 4]. The use of PACs allows invasive monitoring capabilities for practitioners to manage a variety of critical and cardiac conditions. It is imperative that the provider be very skilled in catheter placement as well as comprehending the risks and complications of this invasive monitoring tool.

Complications of the PAC are divided into initial vascular access, insertion technique of the PAC, and maintenance of the PAC in a branch of the pulmonary artery (right or left). Clearly, the incidence of complications relates to operator skill and technique as well as patient status and selection.

With regard to initial vascular access, strong consideration is necessary for route selection. Arterial puncture can occur with an incidence of 2–16% [5]. This complication can result in a hemothorax if the subclavian route is attempted, whereas a carotid artery hematoma may manifest itself if inadvertent carotid injury occurs during internal jugular cannulation. A carotid artery to internal jugular vein arteriovenous fistula has been reported also when inadvertent cannulation of the carotid artery has occurred [5]. Most of these concerns can be mitigated by using ultrasound guidance during vascular access. In fact, a Cochrane Database Review by Brass and colleagues conducted an extensive search ranging from 1966 until 2013 and identified over 730 studies that fit the search criteria; their analysis noted that use of two-dimensional ultrasound rather than classic anatomic landmarks decreased the total complication rate of vascular access by 71% and of inadvertent arterial access by 72% [6]. In addition, success on the first attempt increased by 57% using ultrasound guidance [6].

Arrhythmias constitute the most common complication in relation to PAC insertion. These are usually premature ventricular contractions or nonsustained ventricular tachycardia. Furthermore, catheter resistance, endocarditis, endocardial damage, pseudoaneurysms, and cardiac valve injury are all possible complications of PAC insertion. Rupture of the pulmonary artery is the most devastating complication of PAC maintenance, with a mortality rate reaching approximately 50% [5].

Avoiding serious complications, in the use of a PAC, requires a proactive awareness of potential catheter complications, in particular when unexpected resistance is felt during catheter withdrawal attempts [7, 8]. Patients with dilated cardiac chambers, as in this case, are more prone to catheter kinking and knotting [8]. The larger chamber dimensions allow the catheter considerable opportunities for locomotion thereby enabling coiling, knotting, or kinking. Interestingly, these catheters function fine and it is only during withdrawal attempts or catheter repositioning that the kinking or coiling is suspected, as noted in our case as well [9–11]. Coiling or knotting of the catheter is usually confirmed by radiography. Management techniques for dealing with a kinked catheter can be nonsurgical or surgical. 

*List of Nonsurgical and Interventional/Surgical Options for Bail Out of Kinked and Knotted PAC*

*Nonsurgical*

- Steady and smooth traction.
- Extraction using a larger sheath.
- Extraction using a semistiff or stiff guide wire to straighten the catheter.

*Interventional and/or Surgical*

- Cut-down over insertion site.
- Snare technique and use of the basket under fluoroscopy.
- Contrast venography with balloon venoplasty to correct stenoses.
- Sternotomy and open surgical extraction.
- In cases of fixed catheters from cardiac surgical suture line, cardiotomy is necessary.

It is important that appropriate consultations are employed, in particular vascular surgeons and interventional radiologists. The use of advanced interventional techniques are usually very helpful in correcting intravascular foreign body issues, and, in fact, endovascular methods are the preferred modality if amenable [12–14]. Appropriate fluoroscopy capabilities including percutaneous access from the femoral vein (unilateral or bilateral approach) may be necessary. The use of snaring techniques or endovascular basket catheters as well as balloon venoplasty may be helpful in dilating stenoses, grasping the foreign body and unwinding. It is imperative that a clear understanding of the technique, pitfalls, complications, and rescue solutions is necessary on the part of the practitioner as the continued use of these catheters is employed.
